# Food Insecurity in Hispanic Populations Is Associated with an Increased Risk of Hepatic Steatosis: A Nationally Representative Study

**DOI:** 10.3390/jcm13113206

**Published:** 2024-05-29

**Authors:** Sebastian Niezen, Daniela Goyes, Aarshi Vipani, Ju Dong Yang, Walid S. Ayoub, Alexander Kuo, Michelle T. Long, Hirsh D. Trivedi

**Affiliations:** 1 Department of Medicine, University of Pittsburgh Medical Center, Pittsburgh, PA 15219, USA; niezensf@upmc.edu; 2 Section of Digestive Diseases, Yale School of Medicine, New Haven, CT 06510, USA; danigoyes@gmail.com; 3 Karsh Division of Gastroenterology and Hepatology, Cedars-Sinai Medical Center, 8900 Beverly Blvd, Los Angeles, CA 90048, USA; aarshi.vipani@cshs.org (A.V.); judong.yang@cshs.org (J.D.Y.); walid.ayoub@cshs.org (W.S.A.); alexander.kuo@cshs.org (A.K.); 4 Comprehensive Transplant Center, Cedars-Sinai Medical Center, Los Angeles, CA 90048, USA; 5 Section of Gastroenterology, Evans Department of Medicine, Boston University School of Medicine, Boston, MA 02118, USA; mtlong@bu.edu

**Keywords:** food security, liver fibrosis, steatotic liver disease, VCTE, metabolic risk factors

## Abstract

**Introduction:** The Hispanic population in the US faces a higher risk of nonalcoholic fatty liver disease (NAFLD). Multiple factors influence this risk, including genetics, environmental factors, and socioeconomic statuses. Inadequate access to nutritious foods, or food insecurity, is prevalent among Hispanic individuals and poses a metabolic risk for both the onset and development of NAFLD. **Materials and Methods:** We utilized the National Health and Nutrition Examination Survey (NHANES) 2017–2020 pre-pandemic data to analyze the association between Hispanic ethnicity, hepatic steatosis, fibrosis, and food insecurity. Vibration-controlled transient elastography (VCTE) was employed to assess liver stiffness (LSM) and controlled attenuation parameter (CAP) scores to determine fibrosis and steatosis, respectively. Linear and ordinal logistic regression models were applied to their continuous, log-transformed, and categorical forms, adjusting for demographics, metabolic comorbidities, and socioeconomic factors. Models were subsequently stratified based on food security statuses. **Results:** A total of 7396 Hispanic participants were included in the study. Under multivariable analysis, Hispanic individuals had higher CAP scores (Beta-coefficient: 10.2 dB/m, 95% CI: 6.1–14.4 dB/m, *p* = 0.001)) vs. non-Hispanic individuals, without statistically significant differences in fibrosis. Food-insecure participants exhibited higher CAP scores than their food-secure counterparts. After stratification, a stronger association between Hispanic ethnicity and CAP scores was evident in the food-insecure group (Beta-coefficient: 11.8 dB/m, 95% CI: 4.4–19.3 dB/m, *p* = 0.003). **Discussion:** This study demonstrates the heightened risk of hepatic steatosis among individuals with Hispanic ancestry in the US. The risk is exacerbated by food insecurity, particularly for Hispanic individuals. The contribution is linked to the dietary habits in this population that lead to metabolic risk factors associated with hepatic steatosis. Considering the rising prevalence of NAFLD and food insecurity, interventions focusing on nutritional support and healthcare access among this population could mitigate these burdens.

## 1. Introduction

The Hispanic population represents one of the most diverse and fast-growing ethnic groups in the United States, comprising 18.7% of the total population (US Census Bureau, 2020). Unfortunately, those with Hispanic ancestry are more prone to the development of nonalcoholic fatty liver disease (NAFLD) and fibrosis compared to individuals of other ethnicities. The prevalence and severity of NAFLD among individuals with Hispanic ancestry are influenced by a multitude of factors, including genetic and environmental factors. For instance, the frequency of the patatin-like phospholipase domain-containing 3 (PNPLA3-148M) allele, which has been strongly associated with hepatic fat content, is higher in those with Hispanic ancestry [1]. Furthermore, cultural and socioeconomic factors, such as dietary and exercise habits, healthcare access, and the impact of living in poverty, may also contribute to these disparities [2].

Food insecurity is a cyclic phenomenon of a poor dietary intake due to an inability to afford healthy foods and a compensatory overconsumption of unhealthy foods, predisposing an individual to increased metabolic risk [3]. Social determinants such as a lack of access to jobs and inadequate wages may contribute to the high rates of food insecurity among individuals with Hispanic ancestry [4]. Prior studies have identified an association between food insecurity and NAFLD-related fibrosis [5]. Furthermore, food insecurity is associated with an increased mortality rate in patients with NAFLD and advanced fibrosis [6]. Hispanic households experience almost twice the rate of food insecurity as the households of non-Hispanic or Latino, white families (19.1% vs. 10%) in the United States [7].

There is a dearth of literature exploring the association of food insecurity with the risk of NAFLD and fibrosis among individuals with Hispanic ancestry [5,6,7]. Hence, our study aims to assess the association between Hispanic ethnicity and hepatic steatosis and fibrosis using vibration-controlled transient elastography (VCTE) in this nationally representative sample. We then evaluate the effect of food security statuses on the association of Hispanic ancestry with NAFLD-related fibrosis and steatosis.

## 2. Material and Methods

### 2.1. Study Design and Population

The National Health and Nutrition Examination Survey (NHANES) is a nationwide cross-sectional survey program released in 2-year cycles that includes individuals considered to be representative of the US population since 1999. The survey is conducted by the National Center for Health Statistics (NCHS) at the Centers for Disease Control and Prevention (CDC) and utilizes a multistage probability with an oversampling design for underrepresented groups to produce a representative cohort of the US’s population. Involved institutions deemed the NHANES database to be de-identified and publicly available, and therefore exempt from Institutional Review Board (IRB) approval. Document consent from all participants was obtained during the time of the NHANES survey being conducted. The survey combines in-person interviews to extract data relevant for demographic representation, health, and dietary patterns, with standardized physical examinations and laboratory tests. Due to the coronavirus disease 2019 (COVID-19) pandemic, the 2-year data were partially collected and combined with the 2017–2018 data to create a 2017-March 2020 pre-pandemic database for accurate population estimates. A more detailed design and rationale has been discussed elsewhere [8].

Recently, updated society guidelines recommend using the new terminology of referring to NAFLD as metabolic dysfunction-associated steatotic liver disease (MASLD). However, for the purpose of this study, we kept the former terminology, as the NHANES definition of NAFLD differs from how the new nomenclature is defined. Future iterations of studies, however, should involve new nomenclature to avoid the unnecessary bias and ambiguity associated with former terminology.

### 2.2. Baseline Characteristics and Food Security

Self-reported information, including participants’ age, sex, race, level of education, family poverty index, diagnosis of diabetes, physical activity, alcohol consumption, and smoking status, were extracted from the questionnaire data. Daily alcohol consumption was dichotomized based on the NAFLD cutoffs (<3 reported drinks per day for women and <4 reported drinks per day for men) for moderate and heavy drinkers. The family monthly poverty level index was calculated and categorized by the ratio of total family income to poverty equivalent income determined by the Department of Health and Human Services poverty guidelines. Data on food security was also extracted from the questionnaire portion of the survey. Food security was assessed at the household level by the US Food Security Survey Module (US FSSM), composed of 18 items for households with children and 10 for those without children [9]. Food insecurity, defined as the limited availability to acquire nutritionally adequate and safe food items, was determined if the household score was consistent with having low food security [3,4,5] or very low food security (6–10 points). In addition to VCTE measurements, anthropometric variables, such as body weight and height, were obtained from the examination data in order to calculate the body mass index (BMI) using the formula BMI = weight (kg)/height (m)^2^.

### 2.3. The Definition and Measurement of Hepatic Steatosis and Fibrosis

For the 2017-March 2020 pre-pandemic sample, all participants aged 12 and older were eligible for vibration-controlled transient elastography (VCTE) to measure both the liver stiffness measurement (LSM) as a surrogate for fibrosis and the controlled attenuation parameter (CAP) as a surrogate for steatosis. Participants were included if they were able to lie down during the duration of the exam, were not pregnant or presented a negative urine test for pregnancy, did not have an implanted electronic medical device, and did not have lesions or dressings at the site of measurement. We restricted the study population to participants aged 20 years and older, with negative hepatitis B and C serologies, and with complete VCTE exams, indicated by a fasting time of at least 3 h prior to the procedure, a total of 10 or more measurements, and a liver stiffness interquartile range/median < 30%. Both M and XL probes were utilized for participants based on their BMI. The LSMs were categorized based on trial data correlating with histopathological staging [10]. Specifically, LSM values less than 8.2 kPa were considered not significant for fibrosis, LSM values ranging from 8.2 kPa to 9.7 kPa were categorized as indeterminate for fibrosis, LSMs ranging from 9.7 kPa to 13.6 kPa were classified as indicative of advanced fibrosis, and LSM values exceeding 13.6 kPa were considered indicative of cirrhosis. The CAP was categorized using cutoffs of 297 dB/m, 317 dB/m, and 333 dB/m for S1, S2, and S3, respectively [11].

### 2.4. Statistical Analysis

We first compared the demographic characteristics and VCTE measurements between those with non-Hispanic vs. Hispanic ancestries. Non-Hispanic ethnicities included white, Black, and Asian ethnicities, as defined by the NHANES. Pairwise comparison was performed with a chi-squared test and linear regression.

We posteriorly constructed univariable and multivariable linear and ordinal logistic regression models to evaluate the relationship between Hispanic ethnicity, LSMs, and the CAP, treating them as continuous variables and categorical variables based on the literature [11]. Multivariable models were adjusted for covariates known to be associated with cardiometabolic risk factors from prior NHANES studies including age, sex, income, BMI, type 2 diabetes, and education levels [12].

To assess the influence of food insecurity on the relationship between those with Hispanic ancestry and both hepatic fibrosis and steatosis, we performed two separate analyses: (1) We explored food insecurity as a possible confounding variable by adjusting it in the multivariable model in addition to the other a priori selected covariates. (2) We also explored food insecurity as a possible effect moderator through stratification by food security status. As a sensitivity analysis, in order to mitigate the confounding effect of excessive alcohol use, we excluded those who reported daily alcohol consumptions above NAFLD thresholds described previously.

All data analyses were conducted using Stata version 16.1 (StataCorp LLC, College Station, TX, USA), utilizing the sampling weights provided by the NHANES.

## 3. Results

### 3.1. The Risk of Hepatic Steatosis and Fibrosis in Hispanic Populations

A total of 7396 participants had complete VCTE measurements and reported their race/ethnicity. The total population characteristics are demonstrated in Table 1. The analysis was restricted to the population who had reported their food security status, with a total of 6945 participants. From the total population, individuals with Hispanic ancestry had a lower family monthly poverty level index, a lower education level, and higher reported food insecurity per household. Under univariable analysis, people with Hispanic ancestry had a significantly higher CAP (Beta-coefficient: 10.9, 95% CI: 5.5–16.4, *p* = 0.001) compared to non-Hispanic individuals. However, there was no significant association observed with the LSMs. In the multivariable analysis, the association remained significant for the CAP (Beta-coefficient: 10.2 dB/m, 95% CI: 6.1–14.4 dB/m, *p* = 0.001) (Table 2), after adjusting for age, sex, income, BMI, type 2 diabetes, food security, and education levels.

### 3.2. Fibrosis and Steatosis Based on Food Security

The characteristics of the two food security status groups are demonstrated in Table 3. Overall, those with food insecurity had a lower poverty level index, a lower education level, and were predominantly of a Hispanic ethnicity (30%) compared to their counterparts who were food secure. Under univariable analysis, individuals of Hispanic ancestry exhibited a significant association with the CAP (Beta-coefficients of 8.4 dB/m, 95% CI: 1.0–15.8 dB/m *p* = 0.02 and 13.1 dB/m, 95% CI: 5.5–20.6 dB/m *p* = 0.002 in those with food security and food insecurity, respectively). However, the association was stronger among those with Hispanic ancestry and food insecurity and remained significant under multivariable analysis (Beta-coefficient: 11.8 dB/m, 95% CI: 4.4–19.3 dB/m, *p* = 0.003) (Table 4). Including an interaction term between Hispanic ancestry and food insecurity did not demonstrate a significant association for the CAP (Beta-coefficient 5.4 dB/m, 95% CI: −3.5–14.2, *p* = 0.2). The association remained significant after excluding those with excessive alcohol use (Beta-coefficient: 13.3 dB/m, 95% CI: 5.5–21.1 dB/m, *p* = 0.002) (Supplementary Tables S1 and S2). No significant association was identified for fibrosis.

## 4. Discussion

Food insecurity is a significant public health concern, particularly among individuals with Hispanic ancestry who are also at risk of developing NAFLD. In this nationally representative study, we confirm prior reports of those with Hispanic ancestry having an associated increased risk of steatosis defined by the CAP score via VCTE. Adding food insecurity status to our multivariable model did not strengthen this association, suggesting a lower predictive power compared to Hispanic ancestry. Upon stratifying the data by food security status, our findings revealed that both food-secure and food-insecure individuals of Hispanic ethnicity exhibited a heightened association with having hepatic steatosis, with the association being more pronounced among those who were food insecure.

Food insecurity leads to the overconsumption of a diet high in calorie-dense, processed foods that contributes to metabolic syndrome, a major risk factor for NAFLD [6]. Furthermore, food insecurity is also a risk factor for sarcopenia and sarcopenic obesity, a condition commonly afflicting those with NAFLD [13,14]. Those of a Hispanic ethnicity are more prone to food insecurity and have an increased risk of NAFLD compared to other populations [15,16]. To our knowledge, this is the first study to demonstrate the impact of food insecurity on Hispanic individuals’ risk of NAFLD prevalence in a nationally representative US-based sample.

Food insecurity is growing in prevalence within the US and may partially mediate the risk of NAFLD development in certain high-risk populations. According to recent global estimates, chronic liver disease affected 1.5 billion individuals worldwide in 2017 [17]. Among the various causes of this, NAFLD was found to be the most prevalent, accounting for 60% of cases [17]. The prevalence of NAFLD in North America’s general population is estimated to be around 24%, with hepatic steatosis affecting approximately one in four US residents [18]. Notably, NAFLD is more commonly observed among individuals with Hispanic ancestry, followed by white individuals and Black individuals [17]. The progression from NAFLD to NASH may lead to advanced liver disease, cirrhosis, and hepatocellular carcinoma [19]. NASH is now recognized as the second most frequent reason for liver transplantation in the United States after chronic hepatitis C, and its incidence continues to rise [19].

Based on previous iterations of the NHANES, there has been an apparent rise in the prevalence of food insecurity from 1999 to 2016, most prevalent in Hispanic ethnicities. A recent study showed the crude weighted trends in food insecurity among Hispanic populations increased from 20% to 35% in six years. In comparison, the prevalence of food insecurity among white adults increased from 6% to 13% in the same cohort [20]. Food insecurity has proven to have a detrimental impact on the health outcomes of individuals with NAFLD. In a recent study, individuals with NAFLD and food insecurity had a significantly higher risk of all-cause mortality (HR, 1.46; 95% CI, 1.08–1.97; *p* = 0.01) when compared to those who were food secure after adjusting for demographic and metabolic risk factors [6]. Additionally, another study revealed that food insecurity was associated with a higher mean LSM (6.89 ± 0.40 kPa vs. 5.77 ± 0.14 kPa, *p* = 0.02) for adults aged 50 years and above compared to those under 50 years old [21].

A potential link is constituted with dietary behavior in the United States, as fast food consumption is highly prevalent compared to other countries, increasing the risk of diabetes and cardiovascular disease. In a recent study analyzing the 2017–2018 cycle from the NHANES, researchers evaluated the quantitative impact of fast food consumption on the liver steatosis risk found through VCTE, and found that fast food consumption is independently associated with liver steatosis [22]. Interestingly, there were evident racial and ethnic differences in diet quality in this population, as non-Hispanic white individuals had worse diet qualities measured by the Healthy Eating Index (HEI), a commonly used diet quality score, compared to their Hispanic counterparts. Despite an inverse relationship between food security and diet quality, this association did not reach statistical significance [23].

Those with food insecurity may also be at an increased risk of sarcopenia due to the chronic malnutrition from processed, less nutritious food consumption (37490203). In fact, sarcopenia in cirrhosis is an independent predictor of mortality with an adjusted hazard ratio of 2.36 (95% CI: 1.23–4.53) [24]. In those with NAFLD, sarcopenic obesity poses a significant threat to long-term outcomes [13]. Therefore, mitigating food insecurity serves as a future avenue to reduce the overall burden of sarcopenia in advanced liver disease. The true insight into how food insecurity influences sarcopenia among those with MASLD has yet to be elucidated.

The association between NAFLD and Hispanic ancestry in food-insecure individuals is likely multifactorial. In our study, we accounted for metabolic and demographic components that could negatively impact this association. In a sensitivity analysis, we excluded binge drinkers, which strengthened the association with the CAP in the fully adjusted models (Supplementary Tables S1 and S2). The relationship was held for both food-secure and -insecure cohorts, highlighting a potential relationship between the frequency rather than the quantity of alcohol consumption for the progression of steatosis that was not accounted for in previous studies [25,26]. The power of the frequency of alcohol consumption in the US population is clear, as the exclusion of heavy drinkers decreased the total cohort by one third, maintaining a stronger association. Interestingly, the highest increase was evident in the food-insecure population, where the affordability of alcoholic beverages is questionable. Further analysis would be required to dissect the specific number of drinks consumed per day in each cohort to determine if food insecurity involves alcohol regardless of pricing.

On the other hand, our study did not demonstrate a relationship between Hispanic ethnicity, liver fibrosis, and food insecurity. The natural history of NAFLD is much less predictable than that of other chronic liver diseases. Undoubtedly, a poor nutrient composition promotes excessive liver fat stores and inflammation, representing the first hit in fibrosis progression [9]. However, multiple parallel factors, such as toxins, viruses, and cholestasis, are also implicated in the development and progression of fibrosis in genetically predisposed individuals [2,27]. We hypothesize that multiple insults acting in concert to cause fibrosis might not be present simultaneously in our study population. Additionally, as fibrosis development occurs more gradually compared to the development of steatosis, the cross-sectional nature of the study could have captured a Hispanic population that was at earlier fibrosis stages and a prospective analysis would be required to detect a difference in progression compared to other populations. Lastly, there is likely a small sample of those with advanced fibrosis or cirrhosis in the general US population to truly detect a difference in our study results.

The results must be interpreted within the potential adjustable factors available from the NHANES. One limitation is the cross-sectional nature of the analysis, which could obscure an at-risk population with different manifestations from a prospective standpoint. The NHANES does not provide genomic data, which could have a role in this association. Multiple studies have proven the presence of a genetic component that is associated with fatty liver prevalence and both Native American and Hispanic populations with high prevalences of patatin-like phospholipase domain-containing 3 (PNPLA3; rs738409 C/G, M148I) polymorphisms [28]. Third, multiple covariates including alcohol consumption, smoking status, and physical activity were extracted from the dietary recall data and their interpretation is prone to recall bias. A more granular categorization of these variables would better highlight key differences. Fourth, we did not evaluate the predominance of specific food products consumed in the food-insecure population that have potential to protect against the development of steatosis. Finally, we could not capture the whole NHANES population, as a significant portion of the population did not proceed with a complete VCTE examination, compromising the generalizability of our findings.

In conclusion, we highlight that individuals with Hispanic ancestry in the US have a higher prevalence of steatosis and NAFLD in comparison to other ethnicities. Although a genetic component and the prevalence of metabolic syndrome can influence NAFLD progression, food insecurity is also significantly associated with disease progression in Hispanic populations [28,29]. To our knowledge, this is the first study demonstrating the influence of food insecurity on the association between Hispanic ethnicity and hepatic steatosis in a nationally representative US population using a direct noninvasive measure of hepatic steatosis, namely, VCTE. These results should highlight our healthcare system’s need for implementing reliable screening protocols within food-insecure populations to halt disease progression.

## Figures and Tables

**Table 1:** Demographics from the NHANES of 2017–2020 based on ethnicity.

	Ethnicity ^a^	
	Non-Hispanic ^d^	Hispanic
	N = 5748	N = 1648
**Age** ^b^ (years)	51.5 (17.5)	47.6 (16.2)
**Gender, n (%)**		
Women	2887 (50.2)	852 (51.7)
**Family Monthly Poverty Level Index, n (%) ^c^**		
≤1.3	1361 (29.1)	477 (40.0)
>1.3 to ≤1.85	668 (14.3)	190 (15.9)
>1.85	2654 (56.7)	525 (44.0)
**Education, n (%)**		
Not a high school graduate	711 (12.4)	646 (39.3)
High school graduate	1431 (24.9)	350 (21.3)
Some college or AA degree	1967 (34.3)	435 (26.4)
College or above	1633 (28.4)	214 (13.0)
**Physical Activity (n%)**		
Physically inactive	2430 (55.8)	589 (48.1)
Physically active	1929 (44.2)	635 (51.9)
**Alcohol use (n%)**		
Moderate drinker	4102 (71.4)	1032 (62.6)
Heavy drinker	1646 (28.6)	616 (37.4)
**Smoking status, n (%)**		
Never a smoker	3248 (56.5)	1075 (65.3)
Past smoker	1372 (23.9)	360 (21.9)
Current smoker	1126 (19.6)	212 (12.9)
BMI	29.4 (5.8)	30.1 (6.2)
**BMI, n (%)**		
Underweight	91 (1.6)	10 (0.6)
Normal weight	1542 (27.1)	268 (16.5)
Overweight	1777 (31.2)	612 (37.6)
Obese	2291 (40.2)	738 (45.3)
**Diabetes, n (%)**	826 (14.4%)	254 (15.4%)
**Median CAP (dB/m)**	261.4 (61.9)	275.4 (61.8)
**Steatosis Category (dB/m), n (%)**		
<296	4134 (71.9)	1034 (62.7)
297–316	492 (8.6)	200 (12.1)
317–332	310 (5.4)	114 (6.9)
≥333	811 (14.1)	300 (18.2)
**Median LSM (kPa)**	5.8 (4.5)	5.8 (4.6)
**Liver Stiffness Category (kPa), n (%)**		
<9.7 kPa	5412 (94.2)	1546 (93.8)
≥9.7 kPa & <13.6 kPa	195 (3.4)	58 (3.5)
≥13.6 kPa	141 (2.5)	44 (2.7)
**Food Insecurity**	1817 (33.6)	783 (50.8)

^a^ Data are presented in numbers of total participants.; ^b^ Continuous variables are reported as mean ± SD and categorical variables are reported as observations (percentage prevalence); ^c^ Defined as the ratio of family income to poverty by the Department of Health and Human Services poverty guidelines, adjusted by family size and geographic location; ^d^ Included non-Hispanic white, non-Hispanic Black, and non-Hispanic Asian individuals; BMI, body mass index; CAP, controlled attenuation parameter; kPa, kilopascals.

**Table 2:** The univariable and multivariable models for LSMs (kPa) and the CAP (dB/m) for the NHANES 2017–2020 Hispanic population with complete VCTE examinations *.

	Univariable Coeff. (95% CI) ^a^	*p*-Value	Model 1 ^b^ Coeff. (95% CI)	*p*-Value	Model 2 ^c^ Coeff. (95% CI)	*p*-Value
**LSM**	−0.1 (−0.4–0.3)	0.6	−0.4 (−0.8–−0.0)	0.03	−0.4 (−0.8–−0.0)	0.04
**Log-transformed LSM**	0.0 (−0.0–0.0)	0.9	−0.0 (−0.1–0.0)	0.07	−0.0 (−0.1–0.0)	0.08
**LSM Categories (OR)**	1.1 (0.8–1.6)	0.6	0.9 (0.6–1.5)	0.7	1.0 (0.6–1.5)	0.8
**CAP**	10.9 (5.5–16.4)	<0.001	11.4 (5.8–16.9)	<0.001	11.3 (5.8–16.9)	<0.001
**Log-transformed CAP**	0.0 (0.0–0.1)	<0.001	0.1 (0.0–0.1)	<0.001	0.1 (0.0–0.1)	<0.001
**CAP Categories (OR)**	1.4 (1.1–1.7)	0.002	1.7 (1.3–2.2)	<0.001	1.7 (1.3–2.1)	<0.001

* All coefficients are rounded to the nearest decimal. ^a^ Coefficients are calculated by linear regression for LSMs (kPa) and the CAP (dB/m), and Odds Ratios (OR) by ordinal logistic regression for stiffness categories (kPa) with <9.7 kPa as a reference group and steatosis categories (dB/m) with <297 dB/m as a reference group. ^b^ Model 1 is adjusted for age, sex, income, BMI, type 2 diabetes, physical activity, and education level; ^c^ Model 2 is adjusted for Model 1 and food security.

**Table 3:** Demographics from the NHANES of 2017–2020 based on the reported food security.

	Food Security ^a^	
	Food Security	Food Insecurity
	N = 4345	N = 2600
**Age ^b^**	52.3 (17.4)	47.7 (16.6)
**Gender, n (%)**		
Women	2173 (50.0)	1353 (52.0)
**Race, n (%)**		
Non-Hispanic	3588 (82.6)	1817 (70.0)
Hispanic	757 (17.4)	783 (30.1)
**Family Monthly Poverty Level Index, n (%)**		
≤1.3	627 (16.9)	1211 (55.7)
>1.3 to ≤1.85	477 (12.9)	381 (17.5)
>1.85	2598 (70.2)	581 (26.7)
**Education, n (%)**		
Not a high school graduate	514 (11.8)	743 (28.6)
High school graduate	886 (20.4)	779 (30.0)
Some college or AA degree	1466 (33.8)	815 (31.4)
College or above	1477 (34.0)	259 (10.0)
**Physical Activity (n%)**		
Physically inactive	1929 (57.7)	909 (47.9)
Physically active	1413 (42.3)	990 (52.1)
Alcohol use (n%)		
Moderate drinker	3145 (72.4)	1692 (65.1)
Heavy drinker	1200 (27.6)	908 (34.9)
**Smoking status, n (%)**		
Never a smoker	2691 (62.0)	1358 (52.2)
Past smoker	1097 (25.3)	550 (21.1)
Current smoker	555 (12.8)	692 (26.6)
**BMI**	29.2 (5.2)	30.3 (7.5)
**BMI, n (%)**		
Underweight	53 (1.2)	42 (1.6)
Normal weight	1104 (25.6)	579 (22.5)
Overweight	1467 (34.0)	770 (29.9)
Obese	1685 (39.1)	1186 (46.0)
**Diabetes, n (%)**	598 (13.8)	422 (16.2)
**Median CAP (dB/m)**	263.6 (61.4)	266.8 (63.6)
**Steatosis Category (dB/m), n (%)**		
<296	3081 (70.9)	1756 (67.5)
297–316	381 (8.8)	268 (10.3)
317–332	259 (6.0)	143 (5.5)
≥333	623 (14.3)	433 (16.7)
**Median LSM (kPa)**	5.8 (4.4)	6.0 (4.9)
**Liver Stiffness Category (kPa), n (%)**		
<9.7 kPa	4096 (94.2)	2431 (93.8)
≥9.7 kPa & <13.6 kPa	150 (3.4)	89 (3.5)
≥13.6 kPa	99 (2.5)	80 (2.7)

^a^ Data are presented in numbers of total participants; ^b^ Continuous variables are reported as mean ± SD and categorical variables are reported as observations (percentage prevalence); BMI, body mass index; CAP, controlled attenuation parameter; kPa, kilopascals. All coefficients are rounded to the nearest decimal.

**Table 4:** The univariable and multivariable models for LSMs (kPa) and the CAP (dB/m) with the Hispanic population stratified by food security status with complete VCTE exams *.

	Univariate Coeff B. (95% CI) ^a^	*p*-Value	Model 1 ^b^ Coeff B. (95% CI)	*p*-Value
**Food Security**				
**LSM**	−0.1 (−0.4–0.2)	0.3	−0.3 (−0.8–0.1)	0.09
**Log-transformed LSM**	−0.0 (−0.0–0.0)	0.6	−0.0 (−0.1–0.0)	0.3
**LSM Categories (OR)**	1.0 (0.7–1.6)	0.8	1.1 (0.6–2.0)	0.7
**CAP**	8.4 (1.0–15.8)	0.02	9.8 (3.2–16.4)	0.005
**Log-transformed CAP**	0.0 (0.0–0.1)	<0.001	0.1 (0.0–0.1)	0.002
**CAP Categories (OR)**	1.3 (1.0–1.7)	0.03	1.6 (1.2–2.2)	0.003
**Food Insecurity**				
**LSM**	−0.2 (−0.6–0.3)	0.4	−0.5 (−1.2–0.2)	0.1
**Log-transformed LSM**	−0.0 (−0.0–0.0)	0.6	−0.0 (−0.1–0.0)	0.1
**LSM Categories (OR)**	1.1 (0.6–1.8)	0.7	0.8 (0.4–1.7)	0.5
**CAP**	13.1 (5.5–20.6)	0.002	12.2 (2.8–21.6)	0.01
**Log-transformed CAP**	0.0 (0.0–0.1)	<0.001	0.1 (0.0–0.1)	0.02
**CAP Categories (OR)**	1.4 (1.1–1.8)	0.01	1.3 (0.9–1.9)	0.1

^a^ Coefficients are calculated by linear regression for LSMs (kPa) and the CAP (dB/m), and Odds Ratios (OR) by ordinal logistic regression for stiffness categories (kPa) with <9.7 kPa as a reference group and steatosis categories (dB/m) with <297 dB/m as a reference group.; ^b^ Model 1 is adjusted for age, sex, income, BMI, type 2 diabetes, physical activity, and education level.; * Complete VCTE exam indicated by a fasting time of at least 3 h prior to the procedure, a total of 10 or more measurements, and a liver stiffness interquartile range/median < 30%.

## Supplementary Materials

The following supporting information can be downloaded at: https://www.mdpi.com/article/10.3390/jcm13113206/s1, Table S1: Univariable and Multivariable Models for LSM (kPa) and CAP (dB/m) for NHANES 2017–2020 Hispanic Population with Complete VCTE Examination and <5 reported drinks per day; Table S2: Univariable and Multivariable Models for LSM (kPa) and CAP (dB/m) with Hispanic Population Stratified by Food Security Status with Complete VCTE Exam and <5 reported drinks per day.

## List of Abbreviations

NAFLD	Nonalcohol fatty liver disease
PNPLA3	Patatin-like phospholipase domain-containing 3
VCTE	Vibration-controlled transient elastography
NHANES	National Health and Nutrition Examination Survey
NCHS	National Center for Health Statistics
CDC	Centers for Disease Control and Prevention
COVID-19	Coronavirus disease 2019
US FSSM	United States Food Security Survey Module
BMI	Body mass index
CAP	Controlled attenuation parameter
LSM	Liver stiffness measurement
