# Supplementary material for: Food Insecurity in Hispanic Populations Is Associated with an Increased Risk of Hepatic Steatosis: A Nationally Representative Study
Source: J Clin Med. 2024 May 29;13(11):3206. doi: 10.3390/jcm13113206 (PMC11173297; doi:10.3390/jcm13113206)
Supplement: Supplementary file 1 [file jcm-13-03206-s001.zip › jcm-2971670-supplementary.pdf]

Supplementary Table S1. Univariable and Multivariable Models for LSM (kPa) and CAP (dB/m) for NHANES 2017-2020 Hispanic Population with Complete VCTE Examination and <3 reported drinks per day for women and <4 reported drinks per day for men.*

	**Univariable Coeff B. (95% CI)[a]**	**p-value**	**Model 1[b] Coeff B. (95% CI)**	**p-value**	**Model 2[c] Coeff. (95% CI)**	**p-value**
**LSM**	-0.0(-0.5 – 0.4)	0.8	-0.3 (-0.8 – 0.2)	0.2	-0.3 (-0.8 – 0.2)	0.1
**Log-transformed LSM**	-0.0 (-0.0 – 0.0)	0.9	-0.0 (-0.1 – 0.0)	0.3	-0.0 (-0.1 – 0.0)	0.3
**LSM Categories (OR)**	1.0 (0.7 – 1.6)	0.1	1.0 (0.5 – 1.8)	0.9	1.0 (0.6 – 1.8)	0.8
**CAP**	7.1 (0.4 – 13.9)	0.03	10.2 (3.2 – 17.2)	0.006	10.1 (3.0 – 17.1)	0.007
**Log-transformed CAP**	0.0 (-0.0 – 0.1)	0.05	0.0 (0.0 – 0.1)	0.006	0.0 (0.0 – 0.1)	0.006
**CAP Categories (OR)**	1.3 (1.0 – 1.6)	0.06	1.6 (1.1 – 2.2)	0.01	1.6 (1.1 – 2.2)	0.01

[*] All coefficients B are rounded to nearest decimal.

[a] Coefficients B are calculated by linear regression for LSM (kPa) and CAP (dB/m), and Odds Ratios (OR) by ordinal logistic regression for stiffness categories (kPa) with < 9.7 kPa as reference group and steatosis categories (dB/m) with < 297 dB/m as reference group.

[b] Model 1 is adjusted for age, sex, income, BMI, type 2 diabetes, physical activity, and education level.

[c] Model 2 is adjusted for Model 1 and food security.

**Supplementary Table S2.** Univariable and Multivariable Models for LSM (kPa) and CAP (dB/m) with Hispanic Population Stratified by Food Security Status with Complete VCTE Exam and <3 reported drinks per day for women and <4 reported drinks per day for men.*				
	**Univariate Coeff B. (95% CI)[a]**	**p-value**	**Model 1[b] Coeff B. (95% CI)**	**p-value**
**Food Security**				
**LSM**	-0.2 (-0.6 – 0.2)	0.3	-0.2 (-0.9 – 0.4)	0.4
**Log-transformed LSM**	-0.0 (-0.0 – 0.0)	0.6	-0.0 (-0.1 – 0.0)	0.5
**LSM Categories**	0.9 (0.5 – 1.6)	0.7	1.2 (0.6 – 2.5)	0.6
**CAP**	6.0 (-2.6 – 14.6)	0.1	8.6 (1.5 – 15.7)	0.02
**Log-transformed CAP**	0.0 (0.0 – 0.1)	<0.001	0.0 (0.0 – 0.1)	0.01
**CAP Categories**	1.2 (0.9 – 1.5)	0.3	1.7 (1.3 – 2.3)	0.002
**Food Insecurity**				
**LSM**	-0.6 (-1.2 – 0.1)	0.1	-0.4 (-1.1 – 0.2)	0.1
**Log-transformed LSM**	-0.0 (-0.0 – 0.0)	0.6	-0.0 (-0.1 – 0.0)	0.2
**LSM Categories**	0.9 (0.5 – 1.6)	0.7	0.9 (0.5 – 1.7)	0.6
**CAP**	12.0 (4.0 – 20.0)	0.005	11.5 (-0.3 – 23.3)	0.056
**Log-transformed CAP**	0.0 (0.0 – 0.1)	<0.001	0.1 (-0.0 – 0.1)	0.057
**CAP Categories**	1.3 0.9 – 1.8)	0.1	1.2 (0.7 – 2.1)	0.5

* All coefficients B are rounded to nearest decimal.

a Coefficients B are calculated by linear regression for LSM (kPa) and CAP (dB/m), and Odds Ratios (OR) by ordinal logistic regression for stiffness categories (kPa) with < 9.7 kPa as reference group and steatosis categories (dB/m) with < 297 dB/m as reference group.

b Model 1 is adjusted for age, sex, income, BMI, type 2 diabetes, food security, physical activity, and education level.
